# COVID-19-Associated Dysautonomia

**DOI:** 10.7759/cureus.17156

**Published:** 2021-08-13

**Authors:** Krithika Suresh, Md Didar Ul Alam, Emily Satkovich

**Affiliations:** 1 Internal Medicine, Conemaugh Memorial Medical Center, Johnstown, USA

**Keywords:** orthostatic hypotension, dysautonomia, midordrine, covid-19, syncope

## Abstract

Orthostatic hypotension (OH) refers to a significant reduction in blood pressure (BP) that occurs on standing. It mainly results when autonomic reflexes are impaired or when the intravascular volume is depleted. Symptoms can range from syncope, dizziness, even angina or stroke. Major mechanisms causing OH are autonomic dysfunction affecting the baroreflex, severe volume depletion, and adverse medication effects. Case reports have described neurologic symptom association with coronavirus disease 2019 (COVID-19) including dysautonomia. Although most common symptoms of COVID have been primarily respiratory including fever, cough, shortness of breath, myriad other presentations including neurological, gastrointestinal, cardiac, and thromboembolic presentations have also been described. We describe a patient who was found to have OH and recurrent falls secondary to underlying COVID-19 infection and associated dysautonomia who was successfully treated with midodrine and fludrocortisone.

## Introduction

Orthostatic hypotension (OH) refers to a fall in blood pressure (BP) when standing from a supine position and is highly prevalent in the elderly and those with co-morbidities [1].

It is defined by a drop in BP of at least 20 mmHg for systolic BP and at least 10 mmHg for diastolic BP within three minutes of standing up and is associated with a significant increase in morbidity and mortality. Often multi-factorial, various etiologies can include medication side-effects, autonomic failure due to central or peripheral nervous system disorders (neurogenic OH), and hypovolemia causing non-neurogenic OH [2].

In non-neurogenic OH, treatment of the underlying cause may be curative. Neurogenic OH requires a combination of pharmacologic and non-pharmacologic measures commonly. Patient education and non-pharmacologic measures are initial steps, and fluid repletion and physical counter maneuvers have been proven to be highly effective. Pharmacologic agents consist mainly of drugs that act on blood vessels, blood volume, or with other pressor mechanisms- the most used agents being midodrine and fludrocortisone [3].

## Case presentation

**Figure 1:**
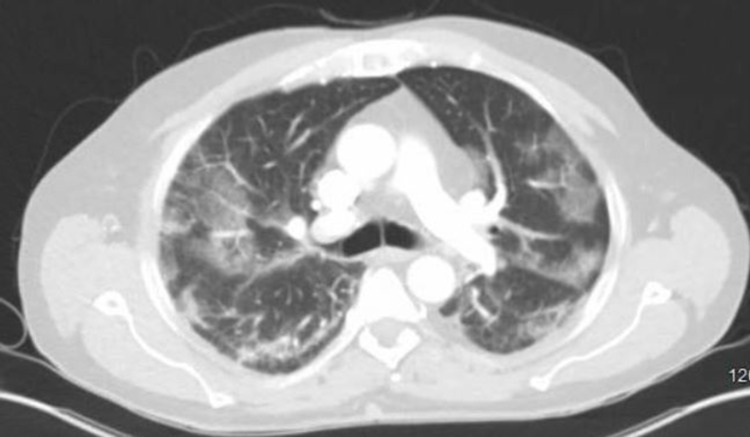
CT chest showing diffuse ground-glass opacities

A 67-year-old male with a past medical history significant for arthritis, benign prostatic hyperplasia (BPH), hyperlipidemia, stroke, and chronic kidney disease presented to the emergency department (ED) with complaints of generalized weakness, lightheadedness, recurrent falls, anorexia, and shortness of breath (SOB). His symptoms began about three days prior to presentation. He initially had a coronavirus disease 2019 (COVID-19) polymerase chain reaction (PCR) test ordered by his primary care provider (PCP) which was positive. Repeat COVID-19 PCR test done in the ED was also positive. He reported increasing weakness with several falls due to weakness. Initial vitals were remarkable for hypoxia with oxygen saturation 84% on room air for which he was placed on 3-liter oxygen via nasal cannula after which his oxygen saturation improved. Initial EKG was largely unremarkable. CT of the head, cervical spine, abdomen, and pelvis done in the ED were negative for acute findings. CT chest showed diffuse ground-glass alveolar opacities consistent with viral pneumonitis (Figure 1).

Initial lab work was also remarkable for creatinine elevated at 1.4 from a baseline of 1.1. He was given a 1-liter normal saline bolus and admitted to the medical floor for further management. He was also started on dexamethasone 6 mg IV daily with symptomatic improvement. However, on day six of hospitalization, the patient had an unwitnessed fall on his face while in the bathroom. He was unsure if he lost consciousness and sustained a nasal laceration and bilateral nasal bone fractures because of this fall. Surgery was consulted and the patient underwent nasal laceration repair. Plastic surgery was consulted for nasal bone fractures and recommended no acute intervention. Due to his recurrent falls and lightheadedness, syncope workup with orthostatic BP, EKG, and echocardiogram were obtained. The echocardiogram revealed an ejection fraction (EF) of 55%-60% without valvular abnormalities. BP was recorded 107/80 while the patient was supine, 95/71 while sitting, and dropped to 66/53 upon standing consistent with OH (Table 1).

**Table 1:** Positive orthostatic vitals recorded bpm-beats per minute

	Supine	Sitting	Standing
Blood Pressure (mmHg)	107/80	95/71	66/53
Heart rate (bpm)	68	70	72

Blood glucose values measured during hospitalization ranged between 90-120 mg/dl essentially ruling out diabetes mellitus. While his orthostatic vitals were being recorded, the patient had a syncopal episode and fell back on his bed and after a few minutes, his BP improved. Cardiology was consulted and he was started on midodrine 5 mg three times daily as well as fludrocortisone 0.1 mg daily. Cardiac monitoring did not reveal any arrhythmia. The patient had improvement in his symptoms; however, on day nine of hospitalization, his orthostatic vitals were still positive and midodrine was increased to 10 mg thrice daily. On day ten of hospitalization, he had a syncopal episode again and was treated with IV fluid bolus and pressure stockings were applied. Fludrocortisone dose was increased to 0.2 mg thrice daily. On day 11 of hospitalization, the patient had no further syncopal episodes; however, he still had positive orthostatic vitals. He was given IV fluids overnight and remained stable the following day after which he was discharged home on a course of midodrine 10 mg thrice daily and fludrocortisone 0.1 mg twice daily, to continue using pressure stockings and with recommendations to follow up with his PCP and neurology. 

## Discussion

In December 2019, pneumonia associated with the severe acute respiratory syndrome coronavirus 2 (SARS-CoV-2) emerged in Wuhan, China, and subsequently spread in many countries becoming a pandemic [4]. Common clinical features consisted mainly of respiratory symptoms and consisted of fever, cough, SOB with the associated radiological feature of interstitial pneumonia on imaging [5]. Although primarily a disease of the respiratory tract, COVID-19 has been found to have a causal association with various neurological and neuropsychological effects [6].

When autonomic reflexes are impaired or intravascular volume is severely depleted, a significant reduction in BP occurs upon standing, a phenomenon termed ‘orthostatic hypotension'. OH, can cause dizziness, syncope, angina, or stroke. The reported prevalence of OH varies with age and the clinical setting [7]. Many disorders can cause OH, with the major mechanisms being autonomic dysfunction affecting the baroreflex, severe volume depletion, and adverse effects of medications [8].

The term dysautonomia refers to a change in autonomic nervous system function that adversely affects health. The changes range from transient, occasional episodes of neurally mediated hypotension to progressive neurodegenerative diseases [9].

Dysautonomia has a wide range of clinical manifestations including, labile BP, OH, impotence, bladder dysfunction, and alteration in bowel functions. Eshak et al, [10] described a case of dysautonomia manifesting as labile BP with varying vasopressor requirements in a patient hospitalized with COVID-19 infection. They also postulated that labile BP could be explained by dysautonomia through afferent baroreflex failure, with OH occasionally present. The key clinical features of afferent baroreflex failure are hypertensive crises, hypotensive episodes, and OH. The mechanism involves damage to the afferent baroreceptor pathway, starting from baroreceptors in the carotid body and finally terminating in the nucleus of tractus solitarius (NTS) [11].

Moreno-Escobar et al, [12] described a case of COVID-19 causing transverse myelitis and dysautonomia manifesting as urinary retention and alteration of bowel function.

Our case was unique as the patient described had improving oxygen requirements, however, was found to have severe OH requiring sympathomimetic agents during hospitalization. He was found to have severe OH despite adequate fluid resuscitation. As this patient had no prior history of neuropathy or neurological disorder and his symptoms were sudden onset after he was diagnosed with COVID-19 infection, we believe that his symptoms largely resulted from COVID-associated-dysautonomia manifesting mainly as severe OH even though our patient lacked other features of autonomic dysfunction such as alteration of bowel or bladder function or erectile dysfunction. The fact that his symptoms improved after he was started on midodrine and fludrocortisone further reiterates that COVID-associated-dysautonomia might largely have been responsible for his symptoms. He was eventually discharged home on a course of midodrine and fludrocortisone.

## Conclusions

Any patient with OH in the presence of underlying COVID-19 infection requires appropriate workup and management of dysautonomia. We treated our patient with midodrine and fludrocortisone with an improvement of his OH. Our literature review did reveal dysautonomia and neurological manifestations being increasingly reported with SARS-CoV-2 infections. 
